# Painful and Prolonged Muscle Cramps following Insulin Injections in a Patient with Type 2 Diabetes Mellitus: Revisiting the 1992 Duke Case

**DOI:** 10.3389/fendo.2017.00243

**Published:** 2017-09-25

**Authors:** Rami A. Ballout, Asma Arabi

**Affiliations:** ^1^Faculty of Medicine, American University of Beirut, Beirut, Lebanon; ^2^Calcium Metabolism and Osteoporosis Program, Department of Internal Medicine, Division of Endocrinology, American University of Beirut Medical Center, Beirut, Lebanon

**Keywords:** insulin aspart, insulin analogs, diabetes, cramps, adverse reaction, case report

## Abstract

A 56-year-old middle-eastern male with a long-standing history of poorly controlled type 2 diabetes mellitus presented to us complaining of severely painful bilateral upper and lower extremity cramps occurring shortly after his rapid-acting insulin analog injection(s). The cramps had started 6 months ago and have been occurring intermittently in non-predictable episodes since then. He had otherwise never experienced any insulin-related adverse reaction(s) before. His cramps are very painful and debilitating, interfering with his daily activities and placing him in a state of constant fear/anxiety of re-experiencing them. This caused him to become non-adherent with his prescribed treatment and poorly compliant with his follow-up regimens. Thorough examination showed a diffuse loss of sensation over the lower limbs. This was subsequently confirmed with a combined electromyography–nerve conduction study which indicated extensive diabetic axonal polyneuropathy. By contrast, lower extremity segmental arterial pressures were negative for peripheral vasculo-occlusive disease, ruling out vascular insufficiency as a possible etiology of the cramps. We then measured the levels of serum electrolytes right-before and 30 min right-after injecting the patient with his insulin. Potassium dropped by about 16% from its initial level, compared to a drop of only around 4% for calcium and none (0%) for magnesium. Thus, we speculated this insulin-induced sharp drop in serum potassium levels as potentiating the patient’s already existing advanced diabetic neuropathy, thereby leading to muscle cramping. However, attempting potassium supplementation for a brief period of time led to a rapid resolution of cramps when they occurred and an overall reduction in their frequency of recurrence. This tilted our diagnosis toward the insulin-induced acute drop in serum potassium levels as the most likely etiology underlying the patient’s cramps. Such an observation has been made only once previously within the literature, back in 1992, at the Duke University Medical Center.

## Introduction

Type 2 diabetes mellitus (T2DM) is initially treated with non-pharmacological interventions of weight loss, exercise, and healthy diet, combined with pharmacotherapeutic agents that enhance insulin sensitivity or responsiveness (1, 2).

However, these, along with other anti-hyperglycemic agents often fail to maintain adequate glycemic control, necessitating the introduction of insulin injections (3).

Nonetheless, subcutaneous insulin injections may themselves sometimes become a source of discomfort for patients, affecting their adherence to treatment and thereby glycemic control (4, 5). This is especially the case in patients experiencing adverse reactions to their insulin injections which can range between the common and well-documented side effects of hypoglycemia (6), weight gain (7), and needle-site cutaneous reactions (edema, erythema, pruritus, and/or lipodystrophy: lipo-atrophy/hypertrophy) (8), to the rare yet severe adversities of fat necrosis (9), hypotension (10), and seizures (11).

We report here the second case in the literature on the development of acute and painful muscle cramps as a rare adversity to insulin injection(s) in a patient with T2DM. We also provide a hypothesis to explain the pathophysiology possibly underlying the cramps.

## Background

A 56-year-old middle-eastern man with an 18-year history of poorly controlled T2DM managed with a basal-bolus regimen of insulin glargine (*Lantus*^®^) and insulin aspart (*Novorapid*^®^), presented with severely painful acute-onset muscle cramps, occurring after his insulin injection(s) for the past 6 months. The cramps occur only after his insulin aspart injections, and not his basal insulin (i.e., insulin glargine).

The patient is an accountant at a finance company, lives with his wife and three kids, and has had no prior surgeries, hospitalizations, and/or accidents. His diet is rich in fats, sugars, and processed foods, and low in fibers. He is also not physical active.

He practices a proper injection technique for his insulin and has never complained of any injection-related complications prior to those 6 months.

His cramps start around 20 min after his dinner-time insulin aspart injection in his anterior thighs bilaterally, migrate posteriorly downward over a 3-min window to his posterior thighs and calves, and then abruptly appear in both hands and feet, causing the fingers and toes to curl inwards. At onset, they are moderately painful in intensity, but become progressively more painful within minutes, eventually making the patient immobile in his bed or chair for up to 3 h sometimes, before they spontaneously resolve.

When they start, the patient usually takes two tablets of a magnesium–B6 complex (*Magne B6*^®^) 100/10 mg, in an attempt to abort them, after he read that magnesium supplementation can reduce muscle cramps. However, this only mildly reduces his cramp pain, without speeding up his recovery.

His fear and anxiety of re-experiencing the cramps have made him poorly compliant with his prescribed therapeutic and follow-up regimens.

On the other hand, when he takes an over-the-counter potassium-rich supplement at the onset of his cramps, as recommended by a distant relative who is a physician, his cramps resolve much faster (in less than 30 min) than they would normally, with minimal, if any, associated pain. He also reports a gradual decrease in the frequency of cramp recurrence following subsequent insulin injections, upon taking the potassium supplement.

Additionally, he complains of several neurological symptoms consistent with his diabetic neuropathy, including persistent paresthesia in both hands with an ulnar nerve sensory pattern (i.e., the radial aspects of both hands with the fourth and fifth digits affected), numbness and diffuse sensory losses over the anterior shins and dorsal and plantar surfaces of the feet, and a reduced sensation along his inner thighs with an erectile dysfunction.

By contrast, the patient denies any intermittent claudication, motor weakness, blurry vision, hypoglycemic episodes, or changes in weight and/or appetite.

On presentation, his blood pressure was 167/78, pulse 89/min, and BMI 28.7 kg/m^2^. Finger-prick glucometer read 460 mg/dL, but he was admitted as he had not taken his medications during that week. HbA1c was ordered on the same day and came out highly elevated attaining 9.9% (85 mmol/mol). Blood pressure measurement upon transitioning from supine-to-standing positions showed no orthostatic hypotension.

Physical examination was overall non-revealing. Sensation was intact in the upper extremities upon pin-prick testing, whereas diffuse loss of gross and discriminative sensations extending from the feet up to the mid-thighs was noted in the lower extremities.

Spinal reflex testing revealed a normal Achille’s reflex on both sides, yet a bilaterally diminished knee-jerk (patellar) reflex. Facial sensation and gross motor power were intact. One month prior to presentation, retinal examination was normal for the right eye, in contrast to the left one which showed neovascularization, an early sign of proliferative diabetic retinopathy. Thus, we counseled our patient to follow-up in 4 months with a repeat retinal examination, as recommended by the American Academy of Ophthalmology (12).

Patient’s family history is significant for T2DM with both of his parents, two of his aunts, and four of his siblings affected by it. His father had also sustained a myocardial infarction and two transient ischemic attacks in his late 50 s. These, added to the recent death of his maternal uncle from an ischemic stroke, merit an extensive assessment of our patient’s cardiovascular status.

In order to investigate the pathophysiology of his cramps, we sought verbal informed consent from the patient to participate in our study, as required by our Institutional Review Board.

We then scheduled a second visit for him, coinciding with his “usual” lunch time. On that visit, we drew blood samples right-before and 30 min right-after his insulin aspart injection, and measured in each sample the serum levels of potassium, calcium, and magnesium. Interestingly, serum potassium levels appeared to have remarkably dropped by around 15.7% from right-before to 30 min after insulin injection, whereas calcium levels had only slightly dropped (around 4.3%), while magnesium levels had not changed (Table 1). Blood glucose had also dropped from 280 mg/dL initially, to around 160 mg/dL at 30 min after insulin injection.

**Table 1:** Electrolyte levels before and after insulin injection.

Sample Electrolytes	S_0_ (before insulin)	S_1_ (after insulin)	Percentage drop (in %) = [(S_0_ − S_1_)/S_0_] × 100
K^+^ (in mmol/L)	5.1	4.3	15.7
Ca^2+^ (in mg/dL)	9.4	9.0	4.30
Mg^2+^ (in mg/dL)	1.9	1.9	0

*Laboratory-specific normal value ranges: K^+^: 3.5–5.1 mmol/L; Ca^2+^: 8.5–10.5 mg/dL; Mg^2+^: 1.6–2.5 mg/dL*.

CBC was ordered and came out normal. However, his HbA1c this time had reached 10.9% (96 mmol/mol), with a serum creatinine level of 1.4 mg/dL (estimated GFR 56 mL/min/1.73 m^2^) and an albumin-to-creatinine ratio of 840.3 mg/g of Cr. His urinalysis also showed remarkable glucosuria (4+ glucose) and proteinuria (1+ proteins). All these findings coupled to his systolic hypertension suggest diabetes-related end-organ damage, specifically, early-stage diabetic nephropathy. This is not surprising in light of his long-standing history of poorly controlled diabetes (13). However, we did not perform any renal ultrasonography to assess kidney size and/or parenchymal width for further confirmation.

We performed a combined electromyography–nerve conduction study (EMG–NCS) whose results confirmed the presence of peripheral diabetic neuropathy, as we had noted on physical examination. Particularly, the EMG–NCS revealed a loss of the sural nerve’s sensory conduction, borderline-low ulnar and median nerve sensory conduction with low-amplitude action potentials in both of them, and a delayed motor conduction in the ulnar and posterior tibial nerves bilaterally. The neurologist interpreting these findings explained that they suggest the presence of extensive peripheral nerve damage, likely resulting from the patient’s long history of poorly controlled diabetes, consistent with diffuse diabetic polyneuropathy.

Finally, we performed an ECG and echocardiography to evaluate the patient’s cardiovascular status, given his previously highlighted high-risk profile. Both came out normal. Normal values for lower extremity segmental arterial pressures were then obtained, negating the presence of any peripheral arterial disease (Table 2).

**Table 2:** Lower extremity segmental arterial pressure test results.

Ankle–brachial index (ABI)	Right	Dorsalis pedis (DP)	1.16
		Posterior tibial (PT)	1.15
	Left	DP	1.19
		PT	1.14
Toe–brachial index (TBI)	Right		1.13
	Left		0.98

## Discussion

Hypoglycemia, weight gain, and needle-site cutaneous reactions are well known as possible complications of insulin therapy that may interfere with either insulin absorption, thereby affecting its efficacy, or a patient’s acceptance for his prescribed treatment, and thereby adherence to it, or both (4, 14).

By contrast, hypotension, seizures, and fat necrosis, though rare, are other possible serious complications of insulin therapy that have been reported in the literature (9–11).

Our patient had never experienced any of the aforementioned adverse reactions to insulin and was rather presenting with a different insulin-related complication that appears to be very rare; he experiences severely painful upper and lower extremity muscle cramps shortly after he injects himself with a rapid-acting insulin analog. This observation has been previously reported only once within the literature, back in 1992 by Meyer and Kirkman at the Duke University Medical Center (15).

In their case however, the patient experienced acute and painful cramps affecting his calves, side thighs, and lower back, around an hour after receiving both: subcutaneous and intravenous regular insulin. This clearly differs from our patient whose lower back was spared but hands and feet (including digits) were affected instead. Moreover, our patient experienced his cramps following only a subcutaneous injection of his rapid-acting insulin analog as opposed to Meyer and Kirkman’s patient whose cramps occurred after receiving both: subcutaneous and intravenous regular insulin injections. Nonetheless, despite the obvious differences in the pattern of cramp occurrence, and the route of insulin used, both patients seem to have experienced muscle cramps in conjunction with their insulin therapy.

In addition to this single published case, we found several online blogs and patient forums addressing insulin-associated muscle cramps (16). It is rather interesting to note that patients on those blogs/forums stated that their muscle cramps occurred specifically in conjunction with their rapid-acting insulin analogs (aspart, lispro, or glulisine), rather than the long-acting formulations (i.e., basal insulin). This is identical to our patient’s reporting, thereby warranting a further investigation of the components of short-acting insulin analogs to possibly identify any constituents likely accountable for those cramps. However, we did not test whether our patient’s cramps would persist with the human regular or rapid-acting insulins, as our patient refused taking any other type of insulin thank his original one, fearing that this may “cause more severe cramps.”

Similar to our investigative approach, Meyer and Kirkman also obtained blood from their patient to analyze his serum electrolyte levels before and after insulin administration, though they focused only on changes in his serum potassium levels (15). Consistent with what we observed in our patient, they too have found serum potassium to have remarkably dropped from the upper-end to the lower-end of its normal range in their patient.

However, for our patient, we also checked for changes in the levels of his serum calcium and magnesium in relation to insulin injection, in addition to potassium. This is due to the extensive literature data on the alteration in the levels of these three electrolytes in patients with T2DM (17). Specifically, chronic hypomagnesemia (18), hypokalemia (19), and/or hypocalcemia (20) have each been reported as a common diabetes-related electrolyte imbalance, with each of them *per se* increasing the propensity to develop muscle spasms.

Given that our patient’s serum potassium level dropped by around 16% within 30 min of his insulin injection, compared with the very small drop in calcium level and no change in magnesium level, we entertained this acute drop in serum potassium level following insulin injection as the likely cause of our patient’s cramps.

In support of our hypothesis, when Meyer and Kirkman infused their patient with dextrose and potassium chloride following his cramp onset, his cramps were rapidly alleviated and had completely resolved in less than an hour. This suggests that the rapid correction of the insulin-induced acute drop in serum potassium levels in their patient through potassium chloride infusion has counteracted the cramps’ etiology, i.e., the rapid drop in potassium levels. In fact, our patient also improved after taking potassium, even faster than Meyer and Kirkman’s patient (30 min compared to 1 h, respectively), possibly because they delivered glucose along with the potassium infusion, which possibly stimulated an additional release of insulin in their patient at first, thereby delaying the recovery of his serum potassium levels.

We also evaluated other possible causes of cramps in the setting of diabetes to check whether any of them could have by itself caused our patient’s cramps, or co-existed as a confounding factor in him and thereby blur our realization of an accurate diagnosis.

While peripheral arterial disease has been widely associated with leg cramps (21), lower extremity segmental arterial pressures in our patient were normal, ruling out any vascular disease as a possible cause of his cramps. On the other hand, an EMG–NCS in our patient showed diffuse diabetes-related sensorimotor polyneuropathy which, according to the neurologist interpreting it having trained on diabetic neuropathy, could be partially responsible for our patient’s cramps. This raised our suspicion of our patient’s diabetic axonal polyneuropathy as a possible confounding factor that is difficult to control for, making us unable to accurately tell whether his cramps resulted only from the acute insulin-induced drop in potassium levels, or rather the combination of the latter with an already existent polyneuropathy potentiating cramp occurrence.

However, the temporal pattern for cramp occurrence, i.e., 15–20 min following insulin injection, with their subsequent resolution rapidly upon potassium supplementation, raises the insulin-induced drop in serum potassium levels as the likely cause of cramps in our patient.

We know that insulin stimulates the Na^+^/K^+^ ATPase in all mammalian cells, inducing an influx of potassium from the extracellular milieu into the cytoplasm (22). However, the reason why only some patients treated with insulin develop a somewhat brief hypokalemic state that leads to cramps remains unclear.

Nonetheless, given that the notion of “hyper-responsiveness to insulin” has been documented and raised elsewhere, it would not be surprising to us if our patient had an underlying mutation that acts to potentiate insulin effects, leading to cramps. Particularly, we hypothesize the presence of a mutation in his Na^+^/K^+^ ATPase that predisposes the latter to hyper-stimulation by insulin with an amplified intracellular potassium shift upon insulin treatment. This then creates a transient hypokalemic state that causes the muscle cramps. However, this remains only a mere speculation from our end and further research is needed to negate or support its validity. In fact, Carrillo et al. had indeed shown an increased affinity of the insulin receptor to its ligand, and a simultaneous elevation in expression and activation levels of the insulin receptor’s downstream signaling mediators, in diabetic and hepatectomized rats (23). The authors of that study founded the notion of the presence of a compensatory “insulin hyper-responsiveness” in patients with diabetes (23). Likewise, although Kung had focused on patients with thyrotoxic periodic paralysis (TPP), he too had spoken of a “hyper-responsiveness to insulin” in TPP patients, implying that such patients “have an underlying predisposition” for an easier activation of their Na^+^/K^+^ ATPase (24).

One may also argue that our patient’s cramps could possibly be a mild form of “insulin neuritis,” a term first coined and described by Caravati in 1933 (25), and subsequently better defined and further characterized by Gibbons and Freeman in 2010 (26). In insulin neuritis, also referred to as “treatment-induced neuropathy of diabetes” (TIND), it is thought that a rapid and intensified anti-glycemic treatment regimen in patients with a previous prolonged history of hyperglycemia in an attempt to attain euglycemia, leads to peripheral small-nerve fiber damage, often with autonomic dysfunction, within 4–10 weeks of treatment (26). However, given that our patient has been taking insulin for around 14 months prior to presentation, i.e., 8 months before he started to experience his cramps, insulin neuritis is unlikely. Moreover, his HbA1c had been persistently high without any remarkable drop in value for over 3 years and has never exceeded 10.9% (most recent value). This further reduces the likelihood of insulin neuritis which often requires a remarkable drop in HbA1c levels, with the lowest pre-treatment HbA1c level ever reported to associate with insulin neuritis being 11.9% (107 mmol/mol) as reported for patient 11 in Gibbons and Freeman’s case series (26). Finally, the absence of any signs or symptoms of autonomic dysfunction in our patient, with the exception of his ED, further refutes the diagnosis of TIND.

Finally, given the lack of data in the literature on the management of such a case and our first-time encounter of it, we referred back to the aforementioned blog searching for patient-attempted interventions that possibly help alleviate the cramps. However, we tried hard not to prescribe any potassium supplementation for our patient, despite that this was previously beneficial for our patient as well as that of the Duke case in aborting the cramps, given its risks of fatal cardiac arrhythmias upon slightly excessive dosing, especially with our patient’s comorbid reduction of kidney function (27).

On the other hand, several patients commenting on the forum reported that taking warm baths during their cramps helped speed-up recovery, with others reporting rapid resolution with quinine water consumption (16). We therefore advised our patient to try warm baths upon cramp onset. We did not recommend he uses quinine water, given the findings of a 2015 Cochrane review that reported low-quality evidence for quinine’s effectiveness in treating nocturnal leg cramps (28), along with the FDA’s 2010 warning against its unapproved use in treating leg cramps where its harmful side effects potentially outweigh any benefits (29).

Our patient now better tolerates his cramps with the help of warm baths, and only very occasionally, takes a low-dose potassium supplement for refractory cramps.

Being intrigued by our observations, we sought written informed consent from our patient to publish his case in a medical journal so that we share his interesting story with the rest of the medical community. Specifically, we would like to address family physicians and endocrinologists taking care of patients with diabetes, as a reminder to keep this rare adversity in mind.

## Concluding Remarks

We hope this case brings into focus the rare yet disabling insulin-related adverse reaction of painful muscle cramps in patients with a long-standing history of diabetes mellitus treated with insulin. Further studies are needed to unravel the pathophysiology underlying such a rare complication, which seemingly affects only a minority of patients receiving insulin for the treatment of diabetes. Such an understanding would allow us to identify the possible factors predisposing to the condition, allowing us to target treatment and provide effective remedies.

## Ethics Statement

This study was exempt from ethical approval procedures being a case report of a single patient who has voluntarily provided oral consent to participate in the study, and written consent to have his case published, for the sake of helping us better understand his case and possibly better manage his symptoms, and share it with the medical community for awareness about it.

## Author Contributions

RB interviewed the patient and obtained full history from him during the patient’s first visit. RB performed the initial physical examination of the patient which was then repeated by AA for confirmation of initial findings. RB saw the patient on his second visit and collected the needed blood samples from him. AA and RB both reviewed the laboratory, nerve conduction study, and peripheral vascular assessment findings for the patient. RB drafted the preliminary copy of the manuscript, and both authors revised and approved the final draft and agreed to be held accountable for the content of this work.

## Conflict of Interest Statement

The authors declare that the research was conducted in the absence of any commercial or financial relationships that could be construed as a potential conflict of interest.
